# High-throughput screening for cell binding and repulsion peptides on multifunctionalized surfaces

**DOI:** 10.1038/s42003-024-06541-7

**Published:** 2024-07-17

**Authors:** Steffen J. Sonnentag, Felix Jenne, Véronique Orian-Rousseau, Alexander Nesterov-Mueller

**Affiliations:** 1 Institute of Biological and Chemical Systems – Functional Molecular Systems, Karlsruhe Institute of Technology, Kaiserstraße 12, 76131 Karlsruhe, Germany; 2 Institute of Microstructure Technology, Karlsruhe Institute of Technology, Kaiserstraße 12, 76131 Karlsruhe, Germany

**Keywords:** Peptides, Screening

## Abstract

The adhesion of cells to the extracellular matrix engages cell surface receptors such as integrins, proteoglycans and other types of cell adhesion molecules such as CD44. To closely examine the determinants of cell adhesion, herein we describe the generation of high-density peptide arrays and test the growth of cells on these multifunctionalized surfaces. The peptide library used consists of over 11,000 different sequences, either random or derived from existing proteins. By applying this screen to SW620 mCherry colorectal cancer cells, we select for peptides with both maximum cell adhesion and maximum cell repulsion. All of these extreme properties are based on unique combinations of amino acids. Here, we identify peptides with maximum cell repulsion on secreted frizzled- and Dickkopf-related proteins. Peptides with strong cell repulsion are found at the poles of the TNF-alpha homotrimer. The formation of cellular patterns on alternating highly repulsive and adhesive peptides are examined. Our screen allows the identification of peptides suitable for biomedical and tissue engineering applications.

## Abstract (web summary)

A screen on high-density peptide arrays identified peptides with high cell-adhesion/ repulsion properties. Peptides with high repulsive properties as those found in TNF-alpha are particularly suitable for biomedical/bioengineering applications.

## Introduction

For most cells, adhesion to a substrate is crucial for survival. Preventing normal cells from adhering will induce cell death, whereas anchorage-independent growth is a property of tumour cells. In normal tissues, epithelial cells are attached to a basement membrane that corresponds to a specialized part of the extracellular matrix (ECM). The ECM, composed of laminins, collagen, fibronectin, growth factors, glycosaminoglycans and other proteoglycans, controls the ability of cells to migrate, differentiate, survive or proliferate^1^. Controlling cell adhesion is therefore a means to control cell behaviour. Accordingly, surface engineering for biomedical applications and tissue engineering rely on the establishment of coatings with adhesive and repellent properties.

A rational way to study cell adhesion is to produce libraries of factors with activating or inhibiting properties that can be tested as coatings for cell attachment. To generate a large number of molecules at a high speed, this approach should be unbiased and efficient, as in the case of ribosomal^2^ or phage displays^3^. The main challenge here is the synthesis of a large number of potential molecules and their transfer to cells.

Currently, there are different techniques for the implementation of such tasks. Most are based on 96-, 384- or 1536-well microplates with varying degrees of automation^4–6^. High-throughput screening (HTS), however, offers a high potential for automation but is accompanied with high costs that only pharmaceutical companies or a few research centres worldwide can afford. Over the past decade, several miniaturized platforms for cell-based assays^7^ have been proposed, such as the encapsulation of cells in droplets formed in an oil phase^8^, SlipChip^9^ or droplet-array sandwiching technology^10^. Miniaturized platforms are very efficient for cell phenotypic and transcriptomic analysis and significantly reduce reagent and cell consumption in comparison to current HTS^11^. Using HeLa-CCL2 cells, it was shown that the same screening experiments as in HTS can be carried out with a significant reduction in culture volumes to 3 nL^12^.

However, volume miniaturization implies a change in cell cultivation conditions, such as pressure, diffusion parameters of the cells, and intensive accumulation of waste products in the limited volume. In addition, сell culture for more than 24 h in nL-scale compartments remains difficult due to evaporation issues and depletion of the cell culture medium.

Transfection on cell microarrays is an alternative approach that consists of pre-spotting transfection mixtures onto a glass slide prior to seeding of cells onto the slide^13,14^. In this approach, cells take up the DNA or RNA on the printed areas, creating spots of localized transfection within a lawn of non-transfected cells. This type of screening made it possible to study cell transfection for many different DNA and RNA oligomer sequences (up to 10,000) without limiting the volume of the cell culture^15^.

Modern high-density peptide arrays synthesized in situ by the combinatorial deposition of amino acids can contain up to a million different peptide spots on a single substrate and thus represent an ideal pool of diverse functional molecules^16–18^. This functionality can be substantially extended by the integration of artificial building blocks^19^, peptide cyclization^20,21^, or peptoid chemistry^22^.

Different short peptides and peptidomimetics, such as Arg-Gly-Asp, either linear or constrained in a cyclic structure, have been generated and extensively studied for their capacity to regulate cell adhesion, migration, self-renewal, and pluripotency^23–27^. Most of these peptides are derived from naturally occurring ECM macromolecules, which can be the source of new synthetic extracellular factors^28,29^. Synthetic ECMs are particularly interesting in that their parameters, such as mechanical properties or permeability, can be more easily tuned.

In the present work, we investigated the possibility of screening for extracellular factors promoting the adhesion or detachment of cancer cells using high-density peptide arrays without limiting the cell culture volume. We explored the functions of peptides at the level of individual amino acids. Among several other interesting sequences, we have identified peptides with extreme cell repulsion on secreted frizzled- and Dickkopf-related proteins.

## Results

### Chip design and experimental setup

**Fig. 1:**
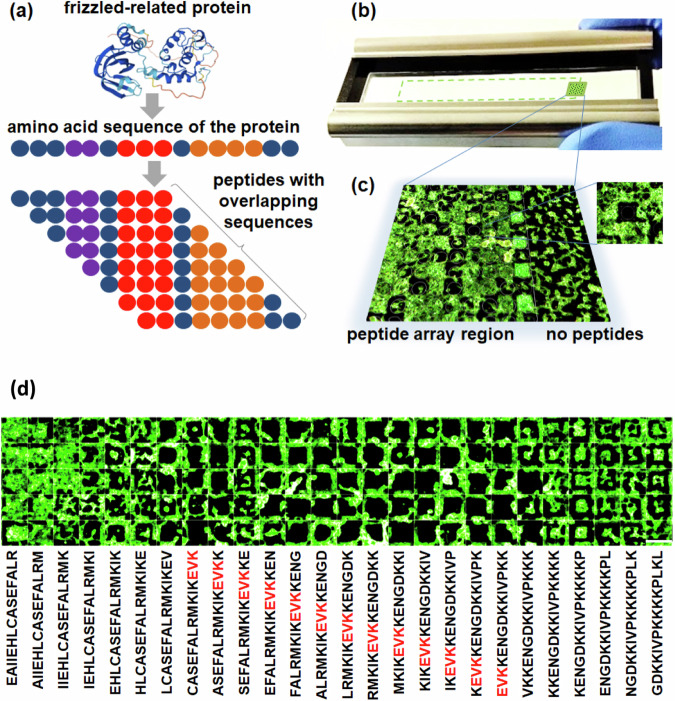
Setup and principle of high-throughput screening of matrix-related peptides with a high-density peptide array. **a** Design of a peptide array from overlapping peptide fragments of functional proteins. **b** Incubation well of a tray with a schematically shown region of the peptide array (dashed green line). **c** Fluorescent image of a peptide array fragment with fluorescently labelled cells. Some peptide pixels exhibit strong cell repulsion (black pixels in the peptide array region). The peptide spot size is 4 × 4 s-pixels (120 µm x 120 µm). **d** Peptide mapping of a selected region of the frizzled-related protein 1 (SFRP1) after incubation with fluorescently labelled cancer cells. Five replicas representing each peptide were arranged in columns. Movement along overlapping peptides shows characteristic cell deposition with good adherence (left), repulsion (middle), or island-like patterns (right). Scale bar: 120 µm.

The peptide library contained 11,314 unique fragments and consisted of three groups of peptides. The first group of peptides was derived from the three proteins secreted frizzled-related protein (SFRP1, Q8N474), Dickkopf-related protein (DKK1, O94907) and tumour necrosis factor (TNF-alpha, Q5STB3). Their sequences were taken from the UniProt database^30^. Five copies of each peptide were presented on the chip. More precisely, this first group consisted of 771 15-mer amino acid sequences derived from overlapping protein sequences differing by one amino acid as illustrated for SFRP1 in Fig. 1a. The second group consisted of 726 substitution sequences derived from four peptides: 5-mers NRWHE (1) and NGWQG (2) and 14-mers KEQWFGNRWHEGYR (1) and QETWFQNGWQGKNP (2), which have been reported to inhibit the co-receptor function of CD44v6 for the receptor tyrosine kinase MET in a human (1) or murine (2) background and thereby inhibit pancreatic tumour growth and metastasis^31^. Substitutions corresponded to a replacement of one amino acid at each position with the remaining 19 biogenic amino acids. Three copies of each peptide from the second group were plated. The most numerous third group of peptides was represented by 9818 15-mer sequences based on random combinations of peptide fragments from the first group. Two copies of each peptide from the third group were plated. In addition, blank spots and human influenza haemagglutinin HA-epitope (YPYDVPDYA) spots were placed on the chip as controls. HA-epitope was used to control the quality of the peptide chip by incubating it with labelled anti-HA antibodies^32^.

The peptide library was synthetized via the high-density peptide microarray technology of axxelera^33^ (Karlsruhe, Germany). The technical minimum synthesis area (a synthesis pixel or s-pixel) was 30 µm × 30 µm. The peptides and their copies were arranged randomly on the peptide chip to prevent local effects on cell adhesion. The peptide synthesis area corresponds to 120 µm × 120 µm (4 × 4 s-pixels). Next, the chip was placed either in an incubation tray (Fig. 1b) or in a live cell imaging incubation chamber. Approximately 10^7^ SW620 mCherry colorectal cancer cells transduced with the TOP-GFP construct, reflecting the activity of the Wnt signalling pathway by the expression of GFP, were transferred onto the chip and incubated for 24 hours. After incubation, the chips were analysed using the confocal fluorescence scanner Innoscan 1100 AL (Innopsys, Carbonne, France). A wide range of diverse cellular patterns were observed, from peptide spots with densely packed cells to spots with no cells at all (Fig. 1c).

### Cellular patterns

Figure 1d shows changes in cell adhesion for peptides with an overlap of one amino acid spanning the entire protein sequence. In this way, we identified the EVK motif as a repellent motif for cells. A large number of cell patterns exhibited incomplete filling of peptide spots. Some of them had a well-defined island shape, such as the peptide KENGDKKIVPKKKKP.

**Fig. 2:**
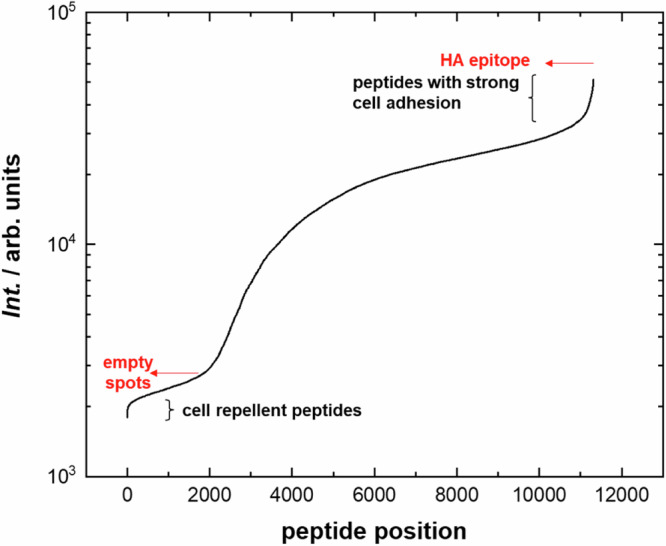
The intensity of the fluorescent signals from cells on peptide spots, arranged in ascending order for the entire peptide library. Red arrows mark the signal from empty spots and from the control HA epitope. Curly brackets at the regions of the curve’s maximum gradients indicate the regions of peptides with specific cell adhesion or repulsion. The intensity of the fluorescent signals is presented on a logarithmic scale.

Figure 2 gives an overview of how the entire peptide library affected cell adhesion. In this graph, all peptides were arranged in ascending order of cell adhesion, as measured by the strength of the fluorescent signal. Although the majority of the curve shows a gradual rise in signal, there are noticeable spikes in the intensity gradient at the curve’s edges.

These regions indicate peptides that evoked either strong repulsion or attraction towards cells.

**Fig. 3:**
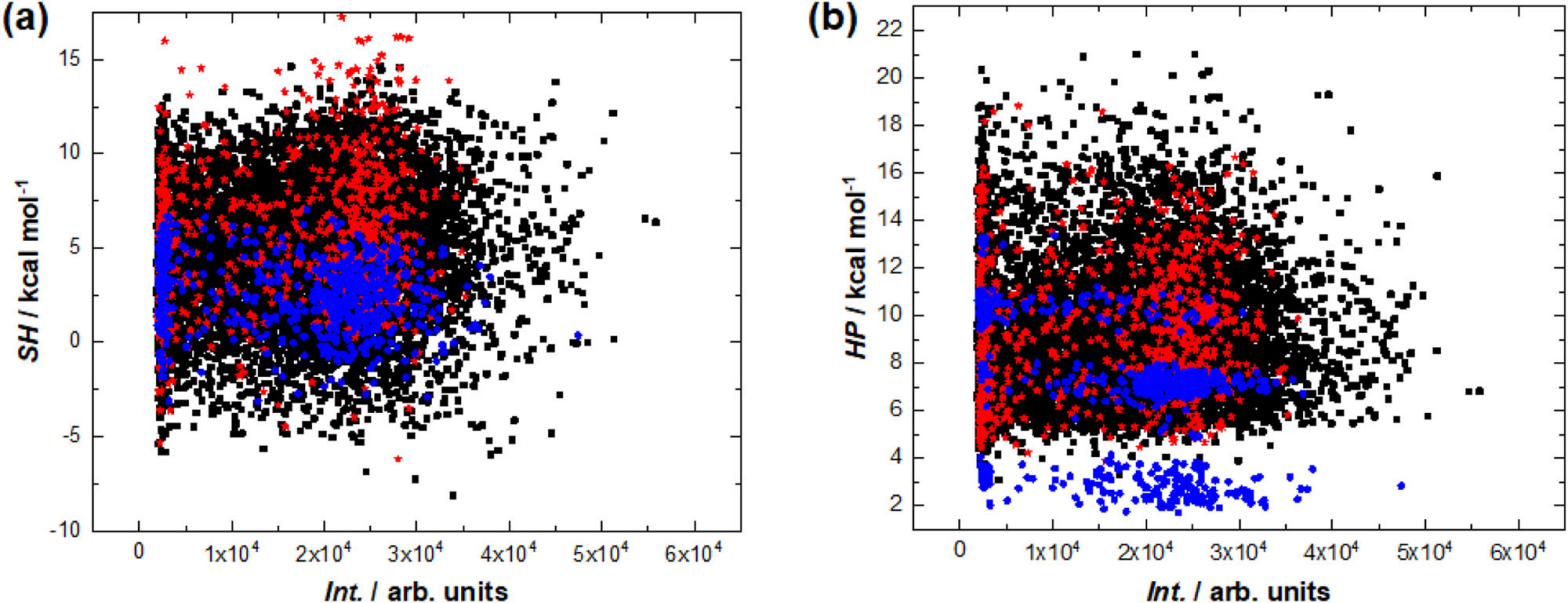
Fluorescent intensity vs. hydrophobicity and helix propensity. Scatter plot: fluorescent intensity *Int.* versus (**a**) the sum of hydrophobicity^46^ *SH* and (**b**) the sum of helix propensity^47^ *HP*. Here and in Supplementary Figs. 1 and 2, the red asterisks, blue circles and black squares indicate the peptides from the three different groups (see result section): overlapping peptides to map proteins, substitutions of special peptides and random peptides, respectively.

To understand how the sequence influences the high adhesion or cell repulsion properties of the peptides, scatter plots reflecting various characteristics of peptides were made: the sum of charges (total charges) (Supplementary Fig. 1), the sum of molecular weights (total molecular weights) (Supplementary Fig. 2), the sum of hydrophobicity (total hydrophobicities) (Fig. 3a) and the sum of helix forming propensity (total helix forming propensity) (Fig. 3b) were calculated. Some of the randomly combined peptides from the third group (black squares) possessed maximum adhesion properties compared to the peptides from the first and second groups (red asterisks and blue circles respectively).

This strong adhesion was not correlated with either the total charge (Supplementary Fig. 1) or the total molecular weight (Supplementary Fig. 2). The independence of the adhesion and the total molecular weight was especially clear in the example of peptides from the second group (blue circles), where peptides with different lengths and thus different molecular weights demonstrated the same adhesion (Supplementary Fig. 2). However, there was a weak trend towards greater total hydrophobicity (Fig. 3a) and lower total helix forming propensity (Fig. 3b) for peptides with strong adhesive properties. The control HA epitope was the peptide with the highest adhesion to cells.

In the region of amino acid sequences with strong cell repulsion (fluorescence intensity tends to zero), the wide distribution of dots over the total hydrophobicity or total helix forming propensity reflects the lack of correlation between the repulsion effect and these properties of the peptides (Fig. 3). Thus, it was necessary to consider each sequence separately.

### Peptides with strong cell repulsion

The first three peptides with the strongest cell-repellent properties were HPGSAVSASNAIKNL, TPPNATEASKPQGTT, and AIKNLPPPTKGQEGS, which are fragments of the DKK1 and SFRP1 proteins. Other peptides of these proteins also appeared in the top ten cell-repellent peptides (Table 1), as well as peptide QETWF.

**Table 1:** List of repulsive peptides on the proteins

N	Protein	Peptide
1	SFRP1	KQQ
2	SFRP1	PNATEASKP
3	SFRP1	EVK
4	SFRP1	AIHKWDKKN
5	DKK1	SVLNSNAIK
6	DKK1	VSAAP
7	DKK1	TLSSKMYHTKGQ
8	DKK1	WSKICKPVLKE
9	TNF-alpha	AEEALPKK
10	TNF-alpha	VRSSSRTP
11	TNF-alpha	SDKKPVAHVVANPQAE
12	TNF-alpha	YQTKV
13	TNF-alpha	PCQRETPEGAEAKP

QNGNQGKN from the second group of the peptide library is a single mutation of the CD44v6-inhibiting peptide QETWFQNGWQGKN, where the amino acid W was replaced by N. In this case, the position of this mutation is important, since the appearance of amino acid N in other positions does not lead to strong cell repulsion (Supplementary Fig. 3). Random combinatorial peptides from the third group, despite their significant numerical superiority in the library, were clearly underrepresented in the group of the strongest cell-repellent peptides.

**Fig. 4:**
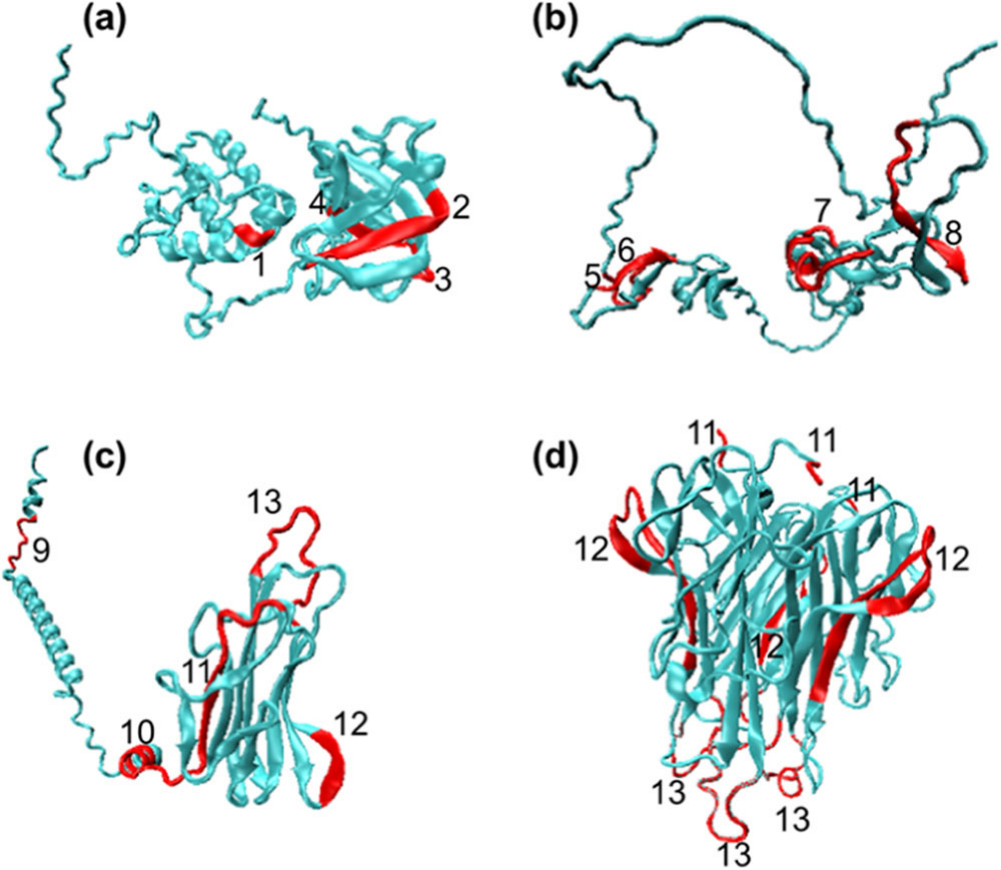
Mapping of cell-repellent peptides on 3D protein models. Cell-repellent peptides are marked in red. **a** Secreted frizzled-related protein 1. **b** Dickkopf-related protein 1. **c** Tumour necrosis factor. **d** Tumour necrosis factor as a homotrimer. The peptides with repulsive properties were clearly expressed at the flexible poles (positions 11 and 13) of the TNF-alpha homotrimer. Numbers represent peptides from Table 1.

We mapped the peptides from Table 1 to the 3D structures of the corresponding proteins, which were predicted using the AI-based tool AlphaFold^34^ (Fig. 4a–d). The first feature of this mapping is that peptides with strong cell repulsion are not associated with a specific secondary structure. They could be located on alpha helixes, beta folds, or fragments with an undefined structure. This was consistent with the results in Fig. 3b, where repulsion did not correlate with the total helix forming propensity. The second feature is that the repulsive peptides in the case of DKK1 and SFRP1 were located in well-defined secondary structures separated by relatively long unstructured flexible amino acid chains (Fig. 4a, b). Since the biologically active secreted form of human TNF-alpha adopts a triangular pyramid shape (Fig. 4c), additional mapping was carried out on an experimentally obtained 3D model of the TNF-alpha homotrimer^35^ (Fig. 4d). Here, the part of the protein containing peptides 9 and 10 was cleaved from the secreted form. The TNF-alpha homotrimer was surrounded by fragments that repel cells. In this case, repulsive peptides 11 and 13 (Table 1) with an undefined structure were located at the trimer poles.

### Formation of cell patterns

**Fig. 5:**
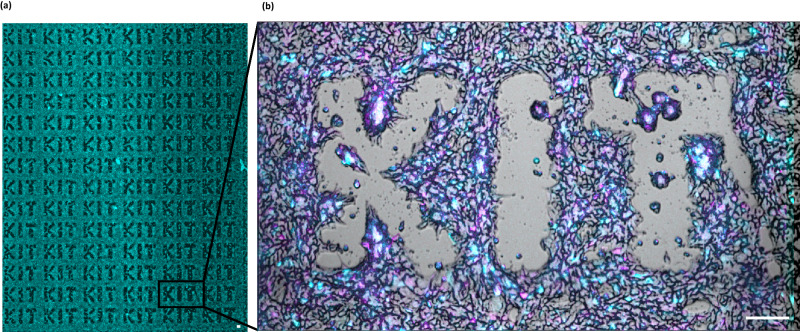
Development of cell patterns on cell-repellent peptides. The pictures and the corresponding video 1 (Supplementary Information) were made with a confocal microscope. SW620 mCherry TOP-GFP cells seeded on the KIT pattern after 24 h. **a** Overview picture of the whole slide. **b** Representative magnification. Magenta: mCherry, turquoise: eGFP. Scale bar: 100 µm.

We examined the settlement of cells on alternating patterns of the cell-repellent and cell-adhesive peptides IAMTPPNATEASKPQ and DRLSAEINRPDYLDF, respectively. Figure 5 shows cell patterns after 24 h of incubation of SW620 mCherry TOP-GFP cells. Peptide patterns were composed in the form of KIT letters, where the letters themselves are the surface coated with a cell-repellent peptide, and the space between the letters is functionalized with a cell-adhesive peptide. The green fluorescence (depicted in turquoise) of the cells originates from the TOP-GFP reporter, reflecting the activated Wnt pathway. The red fluorescence (depicted in magenta) comes from the constitutively expressed mCherry protein.

To follow the formation of such cellular patterns, the behaviour of the cells was examined over time (Supplementary Movie 1, Supplementary Information). Relatively quickly, in the first minutes after seeding, cells were randomly distributed over the entire surface and began to form clusters. After that, the cell clusters left the area of peptides with cell repulsion as can be observed from the formation of the KIT pattern (Fig. 5). The process of cellular clusters leaving the repulsive region can take hours; for example, 24 h as shown in Fig. 5.

**Fig. 6:**
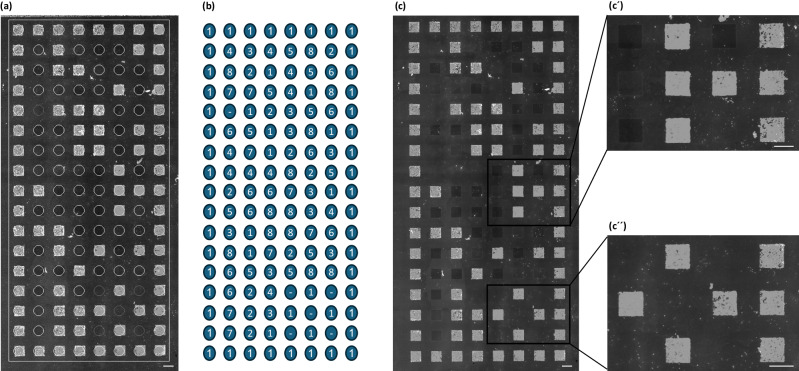
Cellular adhesion on large patterns of cell-repellent or adhesive peptides. **a**, **b** Peptide template and the corresponding blueprint of the printed peptides in areas of 600 µm × 600 µm (20 × 20 s-pixels). **c** Overview picture of the adhered SW620 mCherry TOP-GFP cells after 24 h. Scale bar: 1000 µm. (c´and c´´) Representative enlargements of adhesive or repellent areas. Scale bar: 600 µm.

In order to exclude an influence of the surface area size, we conducted an additional experiment using larger peptide areas corresponding to 600 µm × 600 µm (20 × 20 s-pixels). Here, various peptides selected from the first screen proved to be either repellent (strong and medium) or strongly adherent (Table 2 and Fig. 6a–c). The cells were incubated for 24 h as in the first screen. Within the framework of the variations we used, the properties of peptide spots in repelling or attracting cells turned out to be independent of the size of the spots and their relative position.

**Table 2:** List of representative peptides

N	Property	Peptide
1	Adhesive	YPYDVPDYAG
2	Adhesive	EAIIEHLCASEFALR
3	Adhesive	DRLSAEINRPDYLDF
4	Adhesive	RGD
5	Repulsive	MKIKEVKKENGDKKIV
6	Repulsive	SDKKPVAHVVANPQAE
7	Repulsive	TLSSKMYHTKGQ
8	Repulsive	IAMTPPNATEASKPQ
–	Empty Spot

## Discussion

Our screen allowed the identification of peptides with extreme repulsive and adhesive properties. Cell patterning was demonstrated on minimal structures (120 µm × 120 µm peptide spots corresponding to a spot density of approximately 7000 peptide candidates per square centimetre), as well as on lines with a minimal width of 30 µm (Supplementary Fig. 4). This number of peptides (considering that one microscopic slide has an area of more than 20 cm^2^) makes it possible to implement various cell matrix screening strategies, such as mapping peptides on known proteins (Fig. 1) or substitutional analysis to search for invariant amino acids (Supplementary Fig. 3) at the proteomic scale.

Cell-repellent peptides are certainly useful in modulating cell interactions, as their availability can be controlled through the configuration of the proteins on which they are presented. However, сell-repellent peptides have not been sufficiently studied most likely due to the traditional extensive use of сell-repellent polymers such as poly(ethylene glycol) grafted-poly(L-lysine)^36^ in cell patterning experiments. This gap in cell-repellent peptide research might also be due to the lack of HTS for such peptides.

Of note, the peptides with extreme adhesive properties corresponded to short peptides. This might be due to the fact that protein‒protein binding occurs through the selective recognition of short domains by more complex binding grooves.

We failed to find any common signs of strong adhesion or repulsion for a large number of sequences under consideration. This indicates the high selectivity of the observed interactions. For example, for the CD44v6 14-mer peptide, only a single mutation was critical to significantly enhance its cell repulsion (Supplementary Fig. 3). Most of the peptides with extreme adhesive properties were found on sequences similar to those that actually exist in proteins. A strong cell-attractive HA epitope is present on haemagglutinin, which is responsible for initial viral attachment to receptors on red blood cells^37^ (Figs. 2 and 6, Table 2 Peptide 1).

Most of the cell-repellent peptides we found have not been previously reported in the literature. However, it has been observed that the KQQ motif (Table 1), which functions as a switch, can distinguish between the active and inactive states of nitrogen regulatory protein C^38^. According to our screening, cell-repellent motifs are highly concentrated on secreted proteins, which likely prevents them from non-selectively contacting cells. In this study we have shown for the first time that cell-repellent peptides are located at the poles of the TNF-alpha homotrimer (Fig. 4d).

Due to the restriction of the peptide array technique, the peptide density on the spots throughout the performed experiments could not be changed. It can therefore not be excluded that repulsion was exclusively caused by the peptide primary sequence, or by e.g. the aggregation of peptides. However, the demonstrated screening capability might be a promising first step for rapid initial analysis of peptide-cell interactions and selection of the most promising peptide combinations. The fact that we found cell-repelling peptides at the free ends of the TNF-alpha trimer could be used in favour of the hypothesis of peptide agglomeration for this amino acid sequence. The RGD peptide was synthesized and tested for its cell adhesion on the peptide chip (Fig. 6). However, we did not detect its cell adhesive properties. It is likely that the use of the RGD peptide as a positive control, without an additional spacer to the peptide synthesis surface, is challenging within the framework of our screens. Generally, the cell attraction efficiency of RGD strongly relies on the length and composition of the linker^39,40^. Thus, our technique also offers the possibility to explore such consequences of linker variations, particularly when linear peptides are considered as potential linkers.

The third group based on random combinations of peptide fragments (black dots in Fig. 3) represents the largest group and therefore covers significantly larger functionalities. It clearly contains peptides with the highest adhesion properties compared to those from the first and second groups (red asterisks and blue circles). This may mean that the potential of amino acid sequences with higher adhesion is not exhausted by nature and the development of such peptides may become a promising area of research.

Successful screening of peptides on multifunctional adhesion-modulating surfaces requires control over the rate of formation of cell agglomerates and the intensity of cell migration. Both parameters can be controlled, for example, by cell concentration, temperature and the composition of the cell medium. We observed that some of the clusters that were positioned in the area of repulsion (Supplementary Movie 1, Supplementary Information) did not completely leave this region. This is probably because a sufficiently large part of the agglomerate was positioned in the region of the attracting peptide that the migratory capacity of the cells was not appropriate to either break the agglomerate or move it. Prolonged culturing of cells for approximately ten hours was necessary for maximum removal of agglomerates from the area of cell-repellent peptides. This time may have been necessary to change the expression of surface adhesion molecules, which is induced by cancer cells to enter a mode of collective migration^41^.

Altogether, the proposed method allows rapid screening of peptides with optimal properties for cell experiments. Short peptides, being less expensive, can replace proteins and polymers currently used for cellular patterning. Considering the functional diversity of peptides, it is possible to select specific peptides for single-cell studies. Such peptides could be used in complex cell-confining environments, such as two-state systems with a narrow adhesion gap^42^, to more sensitively quantify the migration of different cancer cell lines^43^.

Invasion by collective migrating carcinomas is known to be characterized by a fine balance between cell‒cell and cell–ECM adhesion^44^. Screening of functional peptides controlling such processes could be performed using our method. In addition, within the framework of this method, it might be possible to use reporter constructs such as TOP-GFP (Figs. 5 and 6), which was used in our system, to detect inhibitors or activators of signalling pathways.

In summary, the uniqueness of the proposed screen lies in its ability to detect potential cell-adhesive/cell-repellent peptides allowing the control of cell behaviour. This method enables the design of surfaces facilitating or blocking growth and migration of mutated cells. This precise patterning of surfaces offers advantages in the field of biomedical engineering (organ on a chip). The identification of cell-repellent peptides in a HTS manner will be of major interest in the medical field especially in the case of vascular stents where they would prevent the adhesion of erythrocytes or thrombocytes. Indeed, one of the main issues in the use of blood-contacting medical devices is the attachment of cells that might result in occlusion of blood vessels. In addition, anti-adhesive agents consisting of natural molecules like peptides would considerably help avoiding intra-abdominal adhesion, a recurrent complication occurring after intestinal surgery.

## Methods

### Cell culture

SW620 mCherry^45^, SW620 mCherry TOP-GFP and HEK293T (American tissue culture collection (ATCC) Cat# CRL-3216, RRID: CVCL_0063, Wesel, Germany) cells were grown in Dulbecco´s Modified Eagle Medium (DMEM) (Gibco^TM^, Thermo Fischer Scientific, Waltham, MA; USA) supplemented with 10% foetal bovine serum (FBS) (Gibco^TM^, Thermo Fischer Scientific, Waltham, MA; USA) and 1% penicillin/streptomycin (Gibco^TM^, Thermo Fischer Scientific, Waltham, MA; USA) (referred to in the following as culture medium) in a humidified incubator (PHC Europe B.V., Etten-Leur, Netherlands) at 37 °C. All experiments were performed using mycoplasma-free cells.

### Lentiviral transduction

Stable transduction of the TOP-GFP reporter was achieved using lentiviral particles produced in HEK293T cells. The envelope plasmid (pVSV-G (2.8 µg)), two packaging plasmids (pRSV-REV (2.5 µg) and pMDLg/pRRE (5 µg)) and the plasmid of interest (TOP-GFP (10 µg) (Addgene, Watertown, MA, USA) were transfected into 80–90% confluent HEK293T cells using PromoFectin (PromoCell, Heidelberg, Germany) according to the manufacturer´s protocol. Six hours after transduction, the culture medium was exchanged. Target (SW620 mCherry) cells were seeded on the same day to reach a confluency of 70% on the day of transduction. 24 h after the culture medium exchange, the supernatant containing the produced virus particles was harvested, filtered through a 0.45 µm filter (Corning, NY, USA) and transferred to the target cells. To increase the number of virus particles, fresh DMEM was applied to virus particle-producing HEK293T cells. 24 h later, the medium was again harvested and filtered as described above. After transduction, the target cells were selected via fluorescence-activated cell sorting (see “Fluorescence-activated cell sorting” section). The cell types were not authenticated.

### Fluorescence-activated cell sorting (FACS)

FACS sorting was performed using a FACSAria^TM^ Fusion Flow Cytometer (BD Biosciences, Heidelberg, Germany). For the selection of a monoclonal cell population containing the TOP-GFP reporter, detachment of the cells was performed using StemPro^TM^Accutase^TM^ (Gibco^TM^, Thermo Fischer Scientific, Waltham, MA; USA), followed by the collection of the cells in serum-containing culture medium. For the analysis and subsequent sorting, 1 × 10^7^ cells were used and resuspended in 3 ml of FACS buffer (2% FBS (Gibco^TM^, Thermo Fischer Scientific, Waltham, MA; USA); 2 mM EDTA (Roth, Karlsruhe, Germany); Dulbecco´s Phosphate Buffered Saline (PBS) (Gibco^TM^, Thermo Fischer Scientific, Waltham, MA; USA)). Monoclonal sorting was achieved using index sorting into a 96-well plate (Greiner-Bio, Frickenhausen, Germany) (1 event per well), followed by reanalysis of the single clones 6 weeks after the initial sorting procedure. Data analysis was performed using FlowJo software (licence number M11c3c353YH92SCS) (BD Biosciences, Heidelberg, Germany).

### Incubation on chips

On the day of incubation, cells were detached using Accutase followed by selection in serum-containing culture medium. Before incubation, the chip was washed with PBS (1% Pen/Strep) and then clamped in a holder provided for this purpose. To incubate the cells on the chip, 1 × 10^7^ cells in 3 ml of DMEM (1% Pen/Strep; 10% FBS; 25 mM HEPES (Roth, Karlsruhe, Germany) were transferred onto the chip and incubated for 24 h either in the incubator or in a live cell imaging incubation chamber (PeCon GmbH, Erbach, Germany). After 24 h, the plates were either analysed using confocal microscopy or fixed.

### Confocal scanning microscopy

24 h after seeding, the slides were analysed using an Innoscan 1100 AL confocal fluorescence scanner (Innopsys, Carbonne, France) with a resolution of 2 µm, PMTGain = 4, excitation wavelength 532 nm, Velocity = 35 l/s. Figure 5 and the corresponding movie were made with a Zeiss LSM 800 confocal microscope.

### Confocal microscopy/live cell imaging

For imaging of the cells, a Zeiss LSM800 confocal microscope (Zeiss, Jena, Germany) was used. Therefore, the cells were seeded as described above. The slide was then transferred to a live cell incubation chamber (PeCon GmbH, Erbach, Germany) (37 °C) and observed for a period of 24 h. Representative pictures and movies were processed using ZEN (Zeiss, Jena, Germany) and iMovie (Apple, Cupertino, CA, USA) softwares.

### Mapping of peptides on the proteins

The simulation tool VMD was used for peptide mapping. 3D protein structures were available in the UniProt database and predicted with AI-based software AlphaFold developed by DeepMind (London, UK). SFRP1: AF-Q8N474-F1; DKK1: AF-O94907-F1; TNF-alpha: AF-Q5STB3-F1. For homotrimer TNF-alpha, the experimental 3D structure was used (PDB 10.2210/pdb1TNF/pdb; Deposition Author(s): Eck, M.J., Sprang, S.R.; Method: X-ray diffraction, Resolution: 2.60 Å).

### Statistics and reproducibility

The graphs in this work represent the fluorescent signal intensity for each peptide spot. This intensity was calculated using the MAPIX program (Innopsys, Carbonne, France). MAPIX calculates the median spot area based on the fluorescence scan and assigns it to the corresponding peptide. If replicate spots for the same peptide were used, then the median was calculated from the intensity values of these spots and assigned to the corresponding peptide for further analysis. The sizes and composition of the peptide libraries, including the number of replicates, are listed in section 2, “Results/Chip Design and Experimental Setup.” The use of replica peptide spots increased the reproducibility of the fluorescent signal. Peptide replicas on the chip were randomly located to avoid bias from local influence on the signal of individual areas of the scanned image. Additionally, in order to test the reproducibility of the extreme adhesive properties of the peptides found during the initial screening, the corresponding peptides were synthesized on different microscope slides and on larger spots (Fig. 6).

### Reporting summary

Further information on research design is available in the Nature Portfolio Reporting Summary linked to this article.

### Supplementary information

Supplementary Information

Description of Additional Supplementary Files

Supplementary Movie

Supplementary Data 1

Supplementary Data 2

Reporting Summary

## Data Availability

The data that support the findings of this study are available as Supplementary Information and from the corresponding authors on request. Tables with fluorescent intensity values after cell incubation for each peptide from libraries synthesized on peptide chips are available in the Zenodo repository, https: https://zenodo.org/records/12604762 (accessed on 1st July 2024). Supplementary Data 1 represents the numerical source data for graphs Fig. 2, Fig. 3, suppl Fig. 1, suppl Fig. 2. Supplementary Data 2 represents the numerical source data for suppl Fig. 3.
