# Supplementary material for: High-throughput screening for cell binding and repulsion peptides on multifunctionalized surfaces
Source: Commun Biol. 2024 Jul 17;7:870. doi: 10.1038/s42003-024-06541-7 (PMC11255233; doi:10.1038/s42003-024-06541-7)
Supplement: Supplementary file 1 — Supplementary Information [file 42003_2024_6541_MOESM1_ESM.pdf]

Supplementary Information for:

High-throughput screening for cell binding and repulsion peptides on multifunctionalized surfaces

Steffen J. Sonnentag[1*], Felix Jenne[2*], Véronique Orian-Rousseau[1§], Alexander Nesterov-Mueller[2§]

1: Institute of Biological and Chemical Systems – Functional Molecular Systems, Karlsruhe Institute of Technology, Karlsruhe, Germany

2: Institute of Microstructure Technology, Karlsruhe Institute of Technology, Karlsruhe, Germany

Supplementary Figure 1:

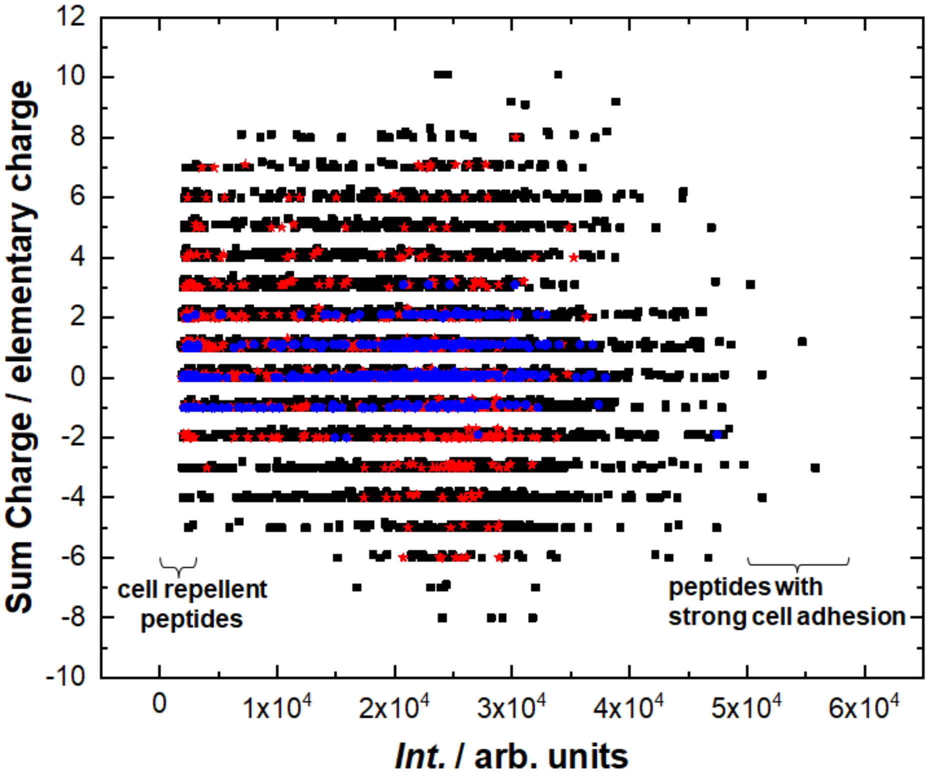

Supplementary Figure 1 | Scatter plot: fluorescent intensity Int. versus the Sum of charges (definition according to D.S. Moore[1]) for each peptide. Here, the red asterisks, blue circles and black squares indicate the peptides from the three different groups (see results section): overlapping peptides to map proteins, substitutions of special peptides and random peptides, respectively.

Supplementary Figure 2:

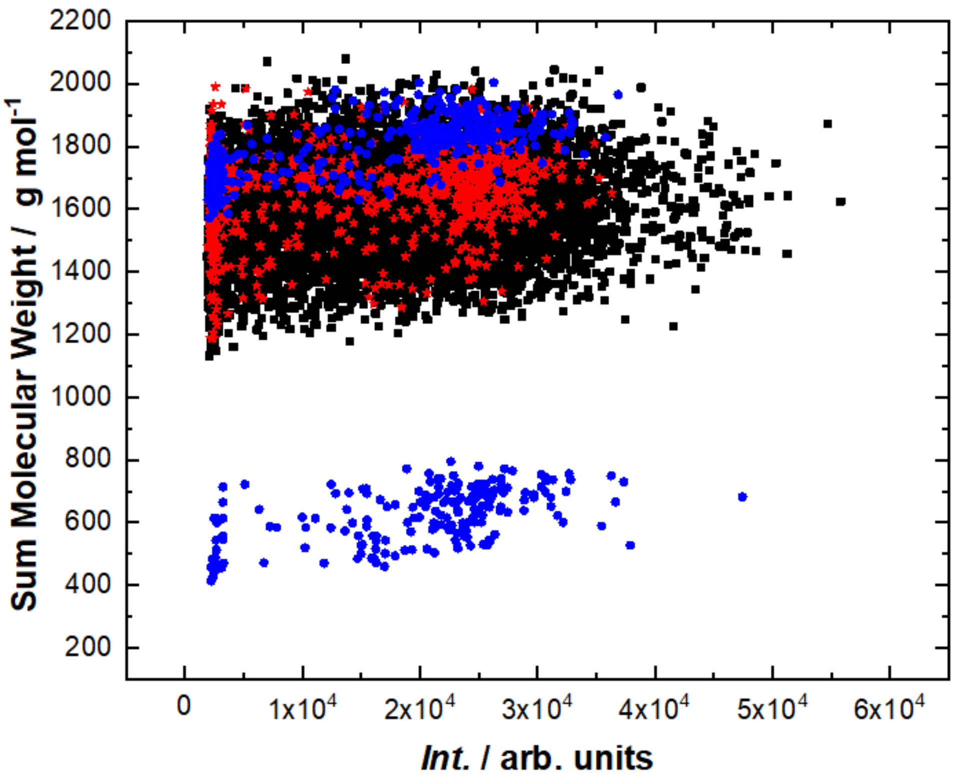

Supplementary Figure 2 | Scatter plot: fluorescent intensity *Int.* versus the *Sum of molecular weight*. Here, the red asterisks, blue circles and black squares indicate the peptides from the three different groups (see result section): overlapping peptides to map proteins, substitutions of special peptides and random peptides, respectively. A separately arranged cluster of low molecular weight (blue circles) corresponds to 5-mer peptides from the substitutional library. The large cluster at the top consists of 15-mer (black squares and red asterisks) and 14-mer peptides (blue circles) from all free peptide groups.

Supplementary Figure 3:

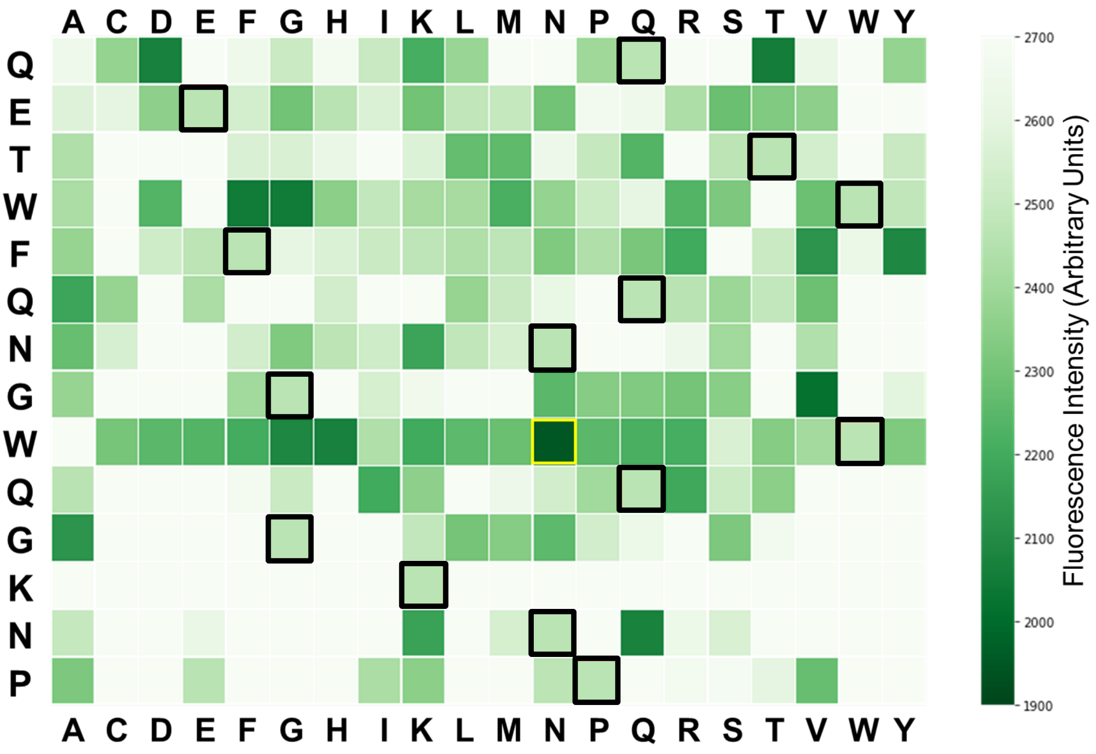

Supplementary Figure 3 | Substitutions for the peptide QETWFQNG<u>W</u>QGKNP. The black squares mark substitution positions. If amino acid W from this peptide is replaced by N, the modified peptide QETWFQNG<u>N</u>QGKNP (yellow square) exhibits maximum cell repulsion.

Supplementary Figure 4:

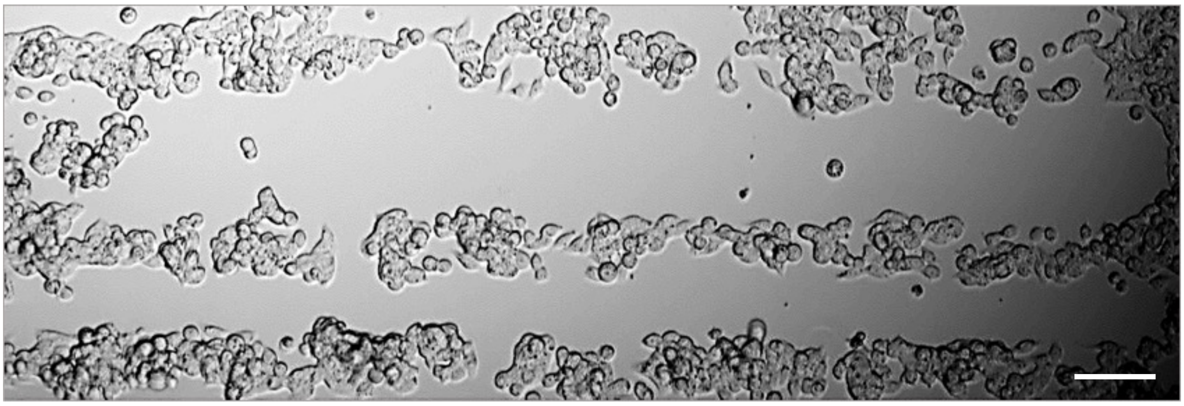

Supplementary Figure 4 | SW620 mCherry TOP-GFP cells were seeded on patterns of cell-repulsive and cell-attracting peptides arranged in lines. The minimal line width is 30 µm, corresponding to the smallest peptide pixel achievable on the chip used. The image was captured after 24 hours of incubation time. Scale bar: 30 µm

References

1 Moore, D. S. Amino acid and peptide net charges: A simple calculational procedure. *Biochemical Education* **13**, 10-11 (1985). https://doi.org:10.1016/0307-4412(85)90114-1
