# Supplementary material for: High-throughput screening for cell binding and repulsion peptides on multifunctionalized surfaces
Source: Commun Biol. 2024 Jul 17;7:870. doi: 10.1038/s42003-024-06541-7 (PMC11255233; doi:10.1038/s42003-024-06541-7)
Supplement: Supplementary file 2 — Description of Additional Supplementary Files [file 42003_2024_6541_MOESM2_ESM.pdf]

Description of Additional Supplementary Files

File Name: Supplementary Movie 1

Description: Live cell imaging of cells on patterns of cell-repellent and adhesive peptides

File Name: Supplementary Data 1

Description: Numerical source data for graphs Fig 2, Fig 3, suppl Fig 1, suppl Fig 2.

File Name: Supplementary Data 2

Description: Numerical source data for suppl Fig. 3.
