# Supplementary material for: High-throughput screening for cell binding and repulsion peptides on multifunctionalized surfaces
Source: Commun Biol. 2024 Jul 17;7:870. doi: 10.1038/s42003-024-06541-7 (PMC11255233; doi:10.1038/s42003-024-06541-7)
Supplement: Supplementary file 6 — Reporting Summary [file 42003_2024_6541_MOESM6_ESM.pdf]

Corresponding author(s): Alexander Nesterov-Mueller
Véronique Orian-Rousseau

Last updated by author(s): Jul 1, 2024

Reporting Summary

Nature Portfolio wishes to improve the reproducibility of the work that we publish. This form provides structure for consistency and transparency in reporting. For further information on Nature Portfolio policies, see our Editorial Policies and the Editorial Policy Checklist.

Statistics

For all statistical analyses, confirm that the following items are present in the figure legend, table legend, main text, or Methods section.

n/a	Confirmed	
☒	☐	The exact sample size (n) for each experimental group/condition, given as a discrete number and unit of measurement
☒	☐	A statement on whether measurements were taken from distinct samples or whether the same sample was measured repeatedly
☒	☐	The statistical test(s) used AND whether they are one- or two-sided<br>*Only common tests should be described solely by name; describe more complex techniques in the Methods section.*
☒	☐	A description of all covariates tested
☒	☐	A description of any assumptions or corrections, such as tests of normality and adjustment for multiple comparisons
☒	☐	A full description of the statistical parameters including central tendency (e.g. means) or other basic estimates (e.g. regression coefficient) AND variation (e.g. standard deviation) or associated estimates of uncertainty (e.g. confidence intervals)
☒	☐	For null hypothesis testing, the test statistic (e.g. F, t, r) with confidence intervals, effect sizes, degrees of freedom and P value noted<br>*Give P values as exact values whenever suitable.*
☒	☐	For Bayesian analysis, information on the choice of priors and Markov chain Monte Carlo settings
☒	☐	For hierarchical and complex designs, identification of the appropriate level for tests and full reporting of outcomes
☒	☐	Estimates of effect sizes (e.g. Cohen's d, Pearson's r), indicating how they were calculated

Our web collection on statistics for biologists contains articles on many of the points above.

Software and code

Policy information about availability of computer code

Data collection	Origin was used for presentation of graphs, Powerpoint was used for the generation of figures
Data analysis	The simulation tool VMD was used for peptide mapping. Monoclonal sorting was achieved using index sorting in a 96-well plate (1 event per well), followed by reanalysis of the single clones 6 weeks after the initial sorting procedure. Analysis of microscopy pictures was performed using Zen software (Zeiss). The movie was generated using iMovie by Apple.

For manuscripts utilizing custom algorithms or software that are central to the research but not yet described in published literature, software must be made available to editors and reviewers. We strongly encourage code deposition in a community repository (e.g. GitHub). See the Nature Portfolio guidelines for submitting code & software for further information.

Data

Policy information about availability of data

All manuscripts must include a data availability statement. This statement should provide the following information, where applicable:

- Accession codes, unique identifiers, or web links for publicly available datasets
- A description of any restrictions on data availability
- For clinical datasets or third party data, please ensure that the statement adheres to our policy

3D protein structures were available in the UniProt database and predicted with AI-based software AlphaFold developed by DeepMind (London, UK). The data that support the findings of this study are available as Supplementary Information and from the corresponding authors on request. Tables with fluorescent intensity

values after cell incubation for each peptide from libraries synthesized on peptide chips are available in the Zenodo repository, https: https://zenodo.org/records/12604762 (accessed on 1st July 2024). Supplementary Data 1 represents the numerical source data for graphs Fig 2, Fig 3, suppl Fig 1, suppl Fig 2. Supplementary Data 2 represents the numerical source data for suppl Fig. 3.

Research involving human participants, their data, or biological material

Policy information about studies with human participants or human data. See also policy information about sex, gender (identity/presentation), and sexual orientation and race, ethnicity and racism.

Reporting on sex and gender	Our research does not involve human participants, their data, or biological material.
Reporting on race, ethnicity, or other socially relevant groupings	Our research does not involve human participants, their data, or biological material.
Population characteristics	Our research does not involve human participants, their data, or biological material.
Recruitment	Our research does not involve human participants, their data, or biological material.
Ethics oversight	Our research does not involve human participants, their data, or biological material.

Note that full information on the approval of the study protocol must also be provided in the manuscript.

Field-specific reporting

Please select the one below that is the best fit for your research. If you are not sure, read the appropriate sections before making your selection.

☒ Life sciences ☐ Behavioural & social sciences ☐ Ecological, evolutionary & environmental sciences

For a reference copy of the document with all sections, see nature.com/documents/nr-reporting-summary-flat.pdf

Life sciences study design

All studies must disclose on these points even when the disclosure is negative.

Sample size	*Describe how sample size was determined, detailing any statistical methods used to predetermine sample size OR if no sample-size calculation was performed, describe how sample sizes were chosen and provide a rationale for why these sample sizes are sufficient.*
Data exclusions	No data were excluded
Replication	In the initial screens, 5 peptide replicas were used.
Randomization	Peptides and their replicas on the chips were located randomly on the chips for initial screens.
Blinding	The investigator did not know the position of the peptide spots, because the positions randomly generated by the peptide synthetizer. The information about the positions was saved in the gal file. The gal file can be used only if fluorescent screen are available after incubation of chips with cells.

Reporting for specific materials, systems and methods

We require information from authors about some types of materials, experimental systems and methods used in many studies. Here, indicate whether each material, system or method listed is relevant to your study. If you are not sure if a list item applies to your research, read the appropriate section before selecting a response.

Materials & experimental systems

n/a	Involved in the study	
☒	☐	Antibodies
☐	☒	Eukaryotic cell lines
☒	☐	Palaeontology and archaeology
☒	☐	Animals and other organisms
☒	☐	Clinical data
☒	☐	Dual use research of concern
☒	☐	Plants

Methods

n/a	Involved in the study	
☒	☐	ChIP-seq
☒	☐	Flow cytometry
☒	☐	MRI-based neuroimaging

Eukaryotic cell lines

Policy information about cell lines and Sex and Gender in Research

Cell line source(s)	SW620 cells were obtained from ATCC
Authentication	No authentication of the cell line was performed
Mycoplasma contamination	Cells were tested every 3 months for mycoplasma. Only myoplasma negative vells were used.
Commonly misidentified lines (See ICLAC register)	N/A

Plants

Seed stocks	No plants were used.
Novel plant genotypes	No plants were used.
Authentication	No plants were used.
